# Supplementary material for: The Effect of Common Inversion Polymorphisms In(2L)t and In(3R)Mo on Patterns of Transcriptional Variation in Drosophila melanogaster
Source: G3 (Bethesda). 2017 Sep 14;7(11):3659–68. doi: 10.1534/g3.117.1133 (PMC5677173; doi:10.1534/g3.117.1133)
Supplement: Supplementary file 1 [file 3659TableS1.docx]

| **Inversion** | Total Genes | Total IAL | IAL Inside Inversion | Genes Inside Inversion | E[X] | p(E[X]>x) |
| --- | --- | --- | --- | --- | --- | --- |
| ***In(2L)t*** | 11969 | 192 | 41 | 1279 | 20 | *4.3045*  *10 ^-6^* |
| ***In(3R)Mo*** |  | 425 | 112 | 871 | 31 | *7.6*  *10 ^-36^* |

Table S1. IAL inside inversion region. Expected counts E[X] and p-values are calculated by hypergeometric distribution with uncorrected p-value cutoff of p=0.025 for the two-tailed test. Genes included in analyses are annotated in both BDGP v5.49 and Affymetrix library version 35.
