# Supplementary material for: The Effect of Common Inversion Polymorphisms In(2L)t and In(3R)Mo on Patterns of Transcriptional Variation in Drosophila melanogaster
Source: G3 (Bethesda). 2017 Sep 14;7(11):3659–68. doi: 10.1534/g3.117.1133 (PMC5677173; doi:10.1534/g3.117.1133)
Supplement: Supplementary file 2 [file 3659TableS2.docx]

| **Inversion** | Total Genes | Total IAL | IAL as *F_st_* outliers | Total *F_st_* outliers | E[X] | p(E[X]>x) |
| --- | --- | --- | --- | --- | --- | --- |
| ***In(2L)t*** | 11969 | 192 | 35 | 1992 | 32 | 0.24089 |
| ***In(3R)Mo*** |  | 425 | 79 |  | 71 | 0.12336 |

Table S2. IAL as *F_st_* outliers in clinal populations. Populations from Reinhardt et al (2014). Number of genes reflect the overlap between the unique Affymetrix Drosophila 2 annotated genes and FlyBase r5.49 annotations. Outliers are from the p=0.05 tail of the empirical distribution of 1kb windows (see Reinhardt et al (2014)).
